# Longitudinal Detection of Tumor-Specific Peptides in Cerebrospinal Fluid for Pediatric Brain Tumor Surveillance

**DOI:** 10.3390/cells15050474

**Published:** 2026-03-05

**Authors:** Kelsi M. Chesney, Jeffrey R. Whiteaker, Brian Hood, Ming Zhou, Huizen Zhang, Samuel Rivero-Hinojosa, Amanda G. Paulovich, Thomas P. Conrads, Brian R. Rood

**Affiliations:** 1 Brain Tumor Institute, Center for Cancer and Immunology Research, Children’s National Research Institute, Washington, DC 20010, USA; kelsichesney1@gmail.com (K.M.C.);; 2 Fred Hutchinson Cancer Center, University of Washington, Seattle, WA 98109, USA; 3 Henry M. Jackson Foundation for the Advancement of Military Medicine, Bethesda, MD 20817, USA; 4 Women’s Health Integrated Research Center, Women’s Service Line, Inova Health System, Annandale, VA 22003, USA

**Keywords:** cerebrospinal fluid, pediatric brain tumors, proteomics, mass spectrometry, tumor-specific peptides, liquid biopsy, longitudinal disease monitoring, neuro-oncology

## Abstract

Pediatric brain tumor survivors remain at high risk of recurrence, yet current surveillance strategies relying on neuroimaging and cerebrospinal fluid (CSF) cytology have limited sensitivity for early or minimal disease. Tumor-specific peptides (TSPs) derived from individual tumors represent a promising class of highly specific biomarkers for longitudinal disease monitoring through CSF-based proteomic analysis. In this study, tumor tissue and serial CSF samples from six pediatric brain tumor patients (five medulloblastomas and one atypical teratoid/rhabdoid tumor (ATRT)) were analyzed using an integrated proteogenomic workflow combining discovery and targeted mass spectrometry. TSPs were identified from resected tumor tissue and matched against shotgun CSF proteomic datasets to nominate candidate biomarkers. High-confidence peptides were synthesized as isotopically labeled standards and quantified longitudinally using targeted multiple reaction monitoring. Two TSP biomarkers derived from individualized pediatric brain tumors (one medulloblastoma and one ATRT) demonstrated robust detection in serial CSF samples and exhibited temporal concordance with radiographic disease course, declining with treatment response and increasing during disease progression. These findings establish the feasibility of detecting and longitudinally quantifying TSPs in CSF and support further investigation of individualized proteomic biomarkers for treatment response monitoring and disease surveillance in pediatric brain tumors.

## 1. Introduction

As treatment methods continue to evolve on the basis of molecular genetics in pediatric brain tumors, survival from initial diagnosis has improved. Whereas the 5-year survival of medulloblastoma ranged from 30 to 50% prior to 2000 [1,2,3], currently up to 85% of patients with average-risk disease achieve long-term survival, though significant differences exist based on risk-stratified subgroups [4,5,6,7,8,9,10,11,12]. Despite modern standardized treatment including maximal safe resection, craniospinal irradiation (CSI), and chemotherapy, approximately 30% of tumors recur [13]. Primary recurrences at any timepoint result in high mortality rates and additionally account for the most significant proportion of late deaths [13,14,15]. In addition to disease-related mortality, cumulative side effects of intensive treatments can affect the quality of life in these patients. One potential way to improve outcomes of recurrent disease would be early detection of recurrence, allowing for early intervention, the success of which may benefit from reduced tumor burden. This remains an untested hypothesis because, until recently, early detection has not been possible. Progress in the field of liquid biopsy is beginning to create opportunities for early detection, but the remaining major challenge is achieving sufficient sensitivity [16,17].

Magnetic resonance imaging (MRI) is the standard method of detecting disease relapses, as it has been shown to detect a majority of recurrences prior to symptom onset [18]. However, the clinical utility of surveillance imaging is debated. Some studies show that surveillance imaging did not provide any survival advantage for patients with detected recurrence [19,20], while other studies report that patients with recurrence detected by MRI have a longer median survival [21]. Asymptomatic patients with disease recurrence are more likely to pursue therapy, which likely contributes to the longer survival times reported in this subgroup [18,21] and highlights the need for highly sensitive detection methods. Recurrent medulloblastoma may be difficult to diagnose on MRI as it does not always enhance with contrast [22,23,24] and may rarely occur at sites outside the central nervous system [25]. Lesions must also be sufficiently large for MRI detection, and bulky disease is presumed to be harder to treat effectively compared to minimal disease. A major challenge lies in distinguishing residual disease from reactive surgical changes on MRI in the early postoperative period. 18F-fluoro-ethyl-tyrosine (18F-FET) PET has been studied in combination with early postoperative MRI in order to increase the specificity of detecting residual tumor in pediatric brain and spinal cord tumors [26]. However, the hybrid PET/MRI system is a challenging setup, and its value diminishes after the 24 h period postoperatively [26]. Additionally, pseudoprogression in the setting of a previously radiated tumor or one treated with immunotherapy is difficult to distinguish from progressive disease on MRI [27].

Cerebrospinal fluid sampling for cytological analysis is used in combination with imaging to increase the detection of recurrent disease. However, only some histologic tumor types can be detected, and CSF cytology is susceptible to inaccuracies for reasons such as post-operative changes, sample processing errors, and low sensitivity [28,29]. When positive, the results are not quantitative and do not allow for definitive staging. Liquid biopsy, a minimally invasive method for monitoring and diagnosing multiple types of cancer through analysis of body fluids, such as blood, sputum, urine, and/or CSF, has great potential in the management of pediatric brain tumors. It can provide molecular information for tumors without invasive tissue sampling, guide treatment decisions, and monitor treatment response [30,31,32,33,34]. Advantages include minimizing morbidity from procedural anesthesia and anatomic complications related to biopsy, as well as molecular profiling that is unbiased by tumor heterogeneity. As liquid biopsies are now starting to be incorporated into clinical trials for pediatric solid tumors (NCT02546453, NCT03496402, NCT03336931), it is crucial that methods are optimized. Cell-free DNA (cfDNA) analysis is one of the most common methods for biomarker detection [32]. As a tool for detecting minimal residual disease (MRD), Liu et al. [17] demonstrated that CSF-derived cfDNA was able to detect recurrence prior to radiographic methods in half of the patients who relapsed with medulloblastoma. When compared to plasma, CSF-derived cfDNA has been shown to be superior for real-time oncologic monitoring for brain tumors due to the intimate contact of CSF with the tumor itself and reduced impact of the blood–brain barrier [30,35].

Complementing genomic analysis, proteomics provides direct insight into the functional and dynamic aspects of cellular processes, allowing for a more detailed understanding of disease mechanisms and paving the way for more personalized and effective cancer treatments. In medulloblastoma, large-scale proteomic analysis of tumor tissue has successfully characterized diagnostic classifications by identifying and quantifying thousands of proteins across different genomic subgroups, providing a more comprehensive understanding of functional tumor biology [36,37,38,39]. Recent large-scale proteogenomic analyses of pediatric brain tumors have incorporated atypical teratoid rhabdoid tumor (ATRT) within multi-histology cohorts, demonstrating distinct proteomic signatures and downstream effects of SMARCB1 loss not fully captured by transcriptomic profiling [37]. However, ATRT remains a rare tumor type, and dedicated proteomic studies focusing specifically on ATRT are comparatively limited. Cerebrospinal fluid represents an attractive substrate for proteomic analysis due to its close contact with central nervous system tumor tissue, likelihood to contain tumor-derived and secreted proteins, and availability for serial sampling to potentially monitor changes in tumor burden associated with treatment response over time.

Here, we describe a specific, precise, and sensitive mass spectrometry-based workflow for proteomic analysis of cerebrospinal fluid (CSF) that integrates discovery proteomics with targeted quantification, with the primary goal of quantifying tumor-specific peptide (TSP) biomarkers unique to the tumors of individual patients. Importantly, TSPs in this context are patient-specific and are not intended to represent shared disease-classification markers across tumor subtypes. The ultimate objective is to enable longitudinal monitoring of tumor-specific and tumor-encoded peptide biomarkers over the progression of a disease, with potential application in early detection of recurrent disease and monitoring response to therapy.

## 2. Materials and Methods

A total of six pediatric brain tumors, of which five were histologically diagnosed as medulloblastoma and one as an atypical teratoid rhabdoid tumor (ATRT), were identified from the Children’s National Brain Tumor Institute Biobank with tumor tissue as well as diagnostic and sequential CSF samples.

TSPs were identified for each individual tumor tissue sample using methods that have been previously described in detail by Rivero-Hinojosa et al. [40]. In summary, the tumor samples were prepared as total protein lysates digested with Trypsin, and high-resolution LC-MS/MS was performed. The resultant spectra were then compared to an individualized tumor database and filtered to exclude results found in healthy cerebellum as well as normal human proteins from the Human NCBI (GRCh38), RefSeq, UniProt Isoforms proteome (UP000005640), neXtProt, and Ensembl (version 84) protein annotations. Peptides originating from a genomic event matching one identified in The Genotype-Tissue Expression (GTEx) RNA-seq collection were also excluded. This proteogenomic filtering strategy was designed to exclude peptides present in canonical human proteomes and non-tumor tissue references, thereby reducing the likelihood of identifying normal physiological or baseline proteins.

### 2.1. CSF Processing

CSF from each of the 6 tumor samples was processed using a Thermo Scientific Pierce Top12 depletion cartridge (Thermo Fisher Scientific, Waltham, MA, USA) to remove abundant serum proteins. The depleted CSF was then buffered in 100 mM triethyl ammonium bicarbonate (TEAB), and disulfide bonds were reduced by the addition of tris(2-carboxyethyl)phosphine (TCEP). Ten microliters of 200 mM TCEP was added to each sample, mixed, and incubated at 55 °C for 1 h. Following reduction, free cysteine residues were alkylated with iodoacetamide to prevent reformation of disulfide bonds. Iodoacetamide (9 mg) was freshly dissolved in 132 µL of 100 mM TEAB immediately prior to use and protected from light. Ten microliters of this solution was added to each sample and incubated for 30 min at room temperature in the dark. Enzymatic digestion was performed with Trypsin (1 µg/µL) at 37 °C overnight. Digestion was halted by the addition of trifluoroacetic acid (TFA) to achieve a final concentration of 0.2%. A 100 µL resin C18 tip was used to desalt each sample with subsequent washing, elution, and mixing. The resulting eluted peptides were then dried in a SpeedVac (Thermo Fisher Scientific, Waltham, MA, USA) and subsequently dissolved in 20 µL QE buffer before being transferred to an autosampler vial.

### 2.2. Discovery Liquid Chromatography–Tandem Mass Spectrometry

Each tryptic-digested CSF sample was desalted and quantified by bicinchoninic acid assay. Samples (1 µg) were loaded on a C18 nano trap column, (Acclaim PepMap100 C18, 2 cm, nanoViper, Thermo Fisher Scientific, Waltham, MA, USA) and resolved on a C18 Easy-Spray column (Acclaim PepMap RSLC C18, 2 µm, 100 Å, 75 µm × 500 mm, nanoViper, Thermo Fisher Scientific, Waltham, MA, USA) with a linear gradient of 2% mobile phase B (95% acetonitrile with 0.1% formic acid) to 32% mobile phase B within 120 min at a constant flow rate of 250 nL/min by nanoflow LC (EASY-nLC 1200, ThermoFisher Scientific, Waltham, MA, USA) coupled online with an Orbitrap Fusion Lumos Tribrid MS (Thermo Fisher Scientific, Waltham, MA, USA). The C18 Easy-Spray column was heated at 50 °C during the analysis. The 12 most intense molecular ions in each MS scan were sequentially selected for high-energy collisional dissociation (HCD) using a normalized collision energy of 35%. The mass spectra were acquired at the mass range of *m*/*z* 400–1600. The Easy-Spray Ion Source (Thermo Fisher Scientific, Waltham, MA, USA) capillary voltage and temperature were set at 2.0 kV and 275 °C, respectively. Dynamic exclusion (15 s) was enabled during the MS2 data acquisition to minimize redundant peptide fragmentation events. The RF lens was set to 30% during the MS analysis, and both MS1 and MS2 spectra were collected in profile mode. Data was searched against a Swiss-Prot human protein database (http://www.uniprot.org/uniprot/ (accessed on 29 October 2021)) using Proteome Discoverer (v.3.0.0.757, Thermo Fisher Scientific, Waltham, MA, USA) via Mascot (v. 2.8.1, Matrix Science Inc., London, UK) with the automatic decoy search option set, followed by false-discovery rate (FDR) processing by Percolator. Data was searched with a precursor mass tolerance of 10 ppm and a fragment ion tolerance of 0.05 Da, a maximum of two tryptic miscleavages, and dynamic modifications for oxidation (15.9949 Da) on methionine residues. Resulting peptide spectral matches (PSMs) were filtered using an FDR of ≤1% (Percolator q-value ≤ 0.01).

### 2.3. Multiple Reaction Monitoring (MRM)

A targeted multiple reaction monitoring (MRM) method was developed by resuspending heavy isotope-labeled peptides in 0.1% formic acid/3% acetonitrile and infusing them into a Thermo Atlis+ triple quadrupole mass spectrometer (TQMS, Thermo Fisher Scientific, Waltham, MA, USA) to identify prominent fragment ions and optimize collision energies. Transitions were optimized for sample 1659 peptide SSNSPHSPIVEEFQVPYNK (720.35 > 521.25, 720.35 > 907.43, 720.35 > 1135.54, 720.35 > 1006.50, 720.35 > 1264.58) and sample 1715 peptide GTFADVTQVT-SLWLAHNEVR (748.72 > 876.97, 748.72 > 934.49, 748.72 > 970.01).

Targeted mass spectrometry on clinical CSF specimens was conducted by resuspending the digested and desalted CSF samples in 20 µL of 0.02% DDM (n-Dodecyl-*β*-D-maltoside, Thermo Fisher Scientific, Waltham, MA, USA, #89902) and spiking in 100 fmol of synthetic isotope-labeled standards. The samples were analyzed by triplicate injections on a Vanquish Neo Liquid Chromatography system (Thermo Fisher Scientific, Waltham, MA, USA) coupled to the Altis+ TQMS using a 0.75 × 150 mm column (Pepmap Neo C18, Thermo Fisher Scientific, MA, USA) and an EasySpray (Thermo Fisher Scientific, Waltham, MA, USA) source. Mobile phases were 0.1% formic acid in water (A) and 0.1% formic acid/80% acetonitrile in water (B), with a gradient of 2–45% B over 17 min at a flow rate of 0.3 µL/min. Dwell times for the MRM method were configured to enable 10 points across the chromatographic peak (minimum dwell time 15 ms). Data were analyzed using Skyline software (v-daily 24.1 [64bit], MacCoss Lab Software, University of Washington, Seattle, WA, USA) [41], and peak area ratios (PAR, light endogenous: heavy isotope-labeled standard) were used for quantification. Lower limits of detection (LLODs) were calculated as ten times the average signal detected across 13 blank runs. The approximate LLODs were 0.02 fmol/µL for peptide GTFADVTQVTSLWLAHNEVR and 0.42 fmol/µL for peptide SSNSPHSPIVEEQVPYNK. Only peptide signals exceeding the established LLOD were reported.

### 2.4. Clinical Data Collection

The electronic medical record was reviewed in a retrospective fashion for all patients with approval of the institutional review board (#0839). Variables collected included date of pathological diagnosis, date of radiographic diagnosis, treatment modalities and dates of treatment, specimens collected (tumor or CSF) and associated dates, radiographic data, and follow-up data. The radiology report for all MRIs performed was reviewed and classified based on the status of the primary lesion: post-operative, no clear evidence of disease, stable size/burden of disease, decreasing size of tumor/less burden of disease, progression of disease, recurrence of disease, or unclear (reactive tissue versus disease process). Follow-up data included the date of the last follow-up, each patient’s status (alive, deceased, or unknown), the most recent tumor status, and prior history of progression or recurrence. When applicable, timelines were created that described the patient’s clinical course, including diagnosis, treatment data, radiographic data, and long-term follow-up information relative to the results of the targeted MRM analysis on serial CSF collections. Reporting of this observational study adheres to the STROBE (Strengthening the Reporting of Observational Studies in Epidemiology) guidelines.

## 3. Results

### 3.1. Tumor-Specific Peptide Discovery from Tumor Tissue

**Figure 1:**
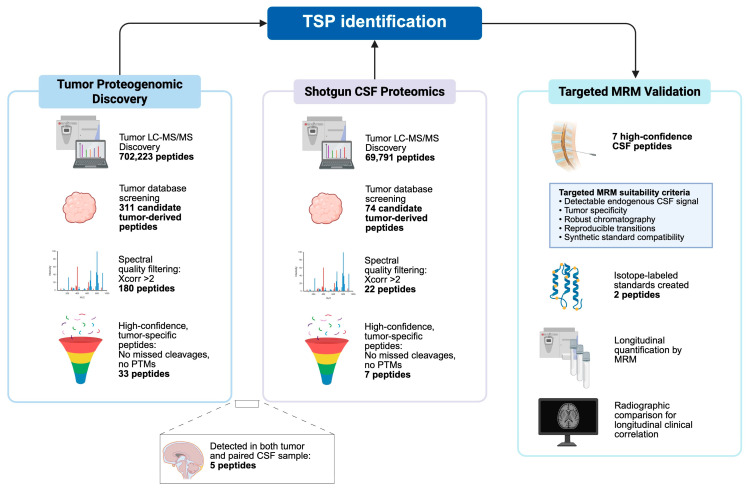
Stepwise tumor-specific peptide identification, filtering, and targeted validation workflow for CSF-based surveillance. Proteogenomic LC-MS/MS of tumor tissue and shotgun CSF proteomics yield large numbers of peptides, the majority of which are host-derived. Sequential tumor-specific filtering and stringent spectral-quality selection nominate a small subset of high-confidence candidates. Only peptides meeting predefined MRM suitability criteria were advanced for isotope-labeled standard synthesis and longitudinal CSF quantification, resulting in two validated tumor-specific biomarkers.

In total, 702,223 peptides were identified across the six tumor tissue samples, of which 311 were tumor-specific. Restricting the dataset to peptides with strong agreement between observed and theoretical spectra and defined by an Xcorr (cross-correlation) value > 2 reduced this set to 180 unique peptides (Table 1, Figure 1). Of these, 14 peptides (7.8%) were found in multiple tumor samples: ten peptides were shared by two samples (5.6%), three across three separate samples (1.7%), and one peptide was identified in four of six tumor samples (LHELSQFK). Of note, the proteogenomic workflow was designed to identify individualized, case-specific tumor-derived peptides, and the majority of TSPs were unique to single patients. As such, peptides detected across multiple cases were not assumed to be disease-specific. To further enrich for the highest-confidence TSPs, peptide lists were further refined by excluding sequences containing post-translational modifications and/or missed cleavages. Following this additional filtering, a final set of 33 peptides was retained as the highest confidence TSPs (Table S1).

### 3.2. Proteomic Analysis of CSF Samples

Shotgun proteomic analysis of CSF samples processed from each tumor type identified 69,791 unique peptides, among which 74 peptides remained after matching against the individualized tumor database and filtering for typical human proteins and normal cerebellum (Table 2). Of these 74 peptides, five were also identified in their corresponding tumor samples. Sample 1659 contained the most matches between tumor and CSF, with four peptides identified on both analyses (SSNSPHSPIVEEFQVPYNK, DQGRVVSAALPRPPPRTGGYR, LTWYLLGPMAAIQMPMLTSSPYTWPIK, ESLLLGAK). Sample 583 identified one match (QRGIDEDDPTADDTSAAVTEEMPPLEGDDDTSRMEEVD) with different modifications on each resultant spectrum. One peptide identified in the analysis of CSF sample 1697 (MSDYNIQKESTLHLVLR) was not found in its corresponding tumor but was identified in the tumor samples 1758 and 1770. Twenty-two peptides from the CSF shotgun analysis demonstrated an Xcorr value > 2. To strictly limit the number of peptides brought forward for subsequent targeted MS analysis, we eliminated peptides with missed cleavages or modifications, leaving six peptides (Table S1).

### 3.3. Targeted Mass Spectrometry Validation of Tumor-Specific Peptides

Targeted multiple reaction monitoring (MRM) analysis was then performed on all CSF samples, and two peptides demonstrated confident identification in two separate patients. Peptide GTFADVTQVTSLWLAHNEVR (Figure S1) was identified in sample 1715, and peptide SSNSPHSPIVEEQVPYNK (Figure S2) in sample 1659. Isotopically labeled synthetic peptides (>85% purity) were generated for each of these sequences by GenScript (Piscataway, NJ, USA) and spiked into serial CSF samples to enable precise relative quantification via MRM.

### 3.4. Longitudinal CSF Peptide Dynamics and Radiographic Correlation

Across the six patients analyzed, longitudinally quantifiable TSPs were validated in two cases. In both patients, TSP abundance changed in temporal concordance with MRI-defined disease course. In this limited cohort, we did not observe TSP elevation clearly preceding radiographic progression, nor were sufficient events available to formally evaluate false-negative TSP results relative to imaging.

**Figure 2:**
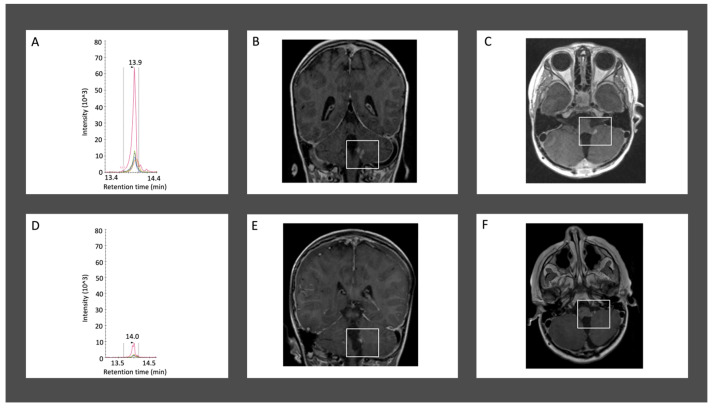
Longitudinal multimodal surveillance demonstrating concordant reduction in CSF endogenous light signal and interval improvement in MRI findings in a treated medulloblastoma patient (1659). (**A**–**C**) correspond to the first post-treatment surveillance timepoint (June 2020) and (**D**–**F**) correspond to the second surveillance timepoint (August 2020). (**A**) Endogenous light signal chromatogram for peptide biomarker SSNSPHSPIVEEQVPYNK from the June 2020 lumbar puncture, showing a prominent peak. (**B**,**C**) Coronal (**B**) and axial (**C**) T1-weighted MRI with contrast demonstrating an enhancing nodule within the left cerebellomedullary fissure (box) at the same June 2020 timepoint. (**D**) Chromatogram from the August 2020 lumbar puncture demonstrating a sharp decline in endogenous light signal for the same peptide marker. (**E**,**F**) Coronal (**E**) and axial (**F**) T1-weighted MRI from August 2020 showing interval reduction in nodularity (box) at the previously visualized site.

**Figure 3:**
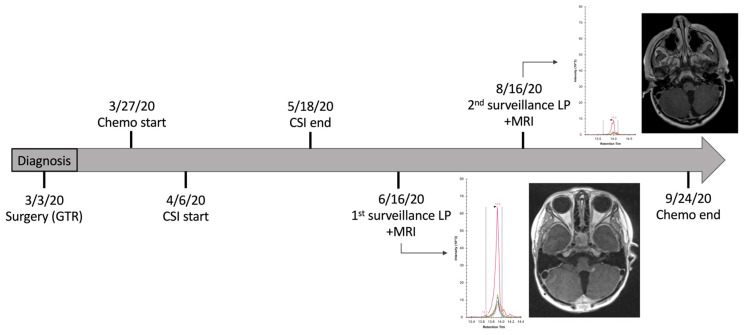
Integrated clinical timeline of treatment, CSF proteomic surveillance, and MRI findings in patient 1659.

Patient 1659 was diagnosed with medulloblastoma on day-of-life (DOL) 1513 and underwent gross total resection of their tumor. Chemotherapy was given DOL 1537–1718, and craniospinal irradiation (CSI) was administered DOL 1547–1589. The endogenous signal for peptide SSNSPHSPIVEEQVPYNK demonstrated a sharp decline between the first surveillance sample (collected DOL 1618; peak area ratio (light:heavy isotopes) (PAR) 19.9 ± 1.4) and a follow-up CSF sample collected two months later (DOL 1679; PAR 0.02 ± 0.0008). This decrease correlated with improvement on MRI, which showed reduced nodularity in the left cerebellomedullary fissure between images obtained on DOL 1617 and DOL 1680 (Figure 2 and Figure 3). The patient’s last imaging obtained on DOL 3413 demonstrated resolution of enhancing nodules and no evidence of disease recurrence. There were no additional CSF samples available for comparative analysis beyond DOL 1679.

**Figure 4:**
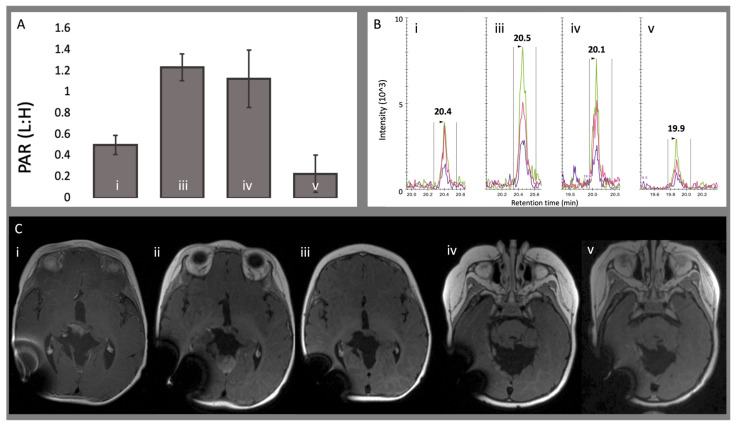
Longitudinal multimodal surveillance demonstrating changes in CSF endogenous light signal and correlated MRI findings in a treated ATRT patient (1715). (**A**–**C**) depict matched surveillance timepoints, with labels i, iii, iv, and v representing dates for which both CSF analysis and MRI were obtained (i = July 2021; iii = February 2022; iv = January 2023; v = April 2024). Subpanel ii corresponds to September 2021, a timepoint with MRI only and no CSF sample collected. (**A**) Peak area ratio (relative to heavy internal standard) demonstrating an increase at timepoint iii, followed by a progressive decline across subsequent surveillance timepoints. (**B**) Endogenous light signal chromatograms showing a parallel increase at timepoint iii with steady reduction thereafter. (**C**) Axial T1-weighted MRI with contrast. (**Ci**) demonstrates residual postoperative tumor. (**Cii**) shows significant interval tumor progression in the subvermian region, prompting escalation of chemotherapy followed by radiotherapy. (**Ciii**) demonstrates a marked reduction in tumor volume, with stability maintained on subsequent follow-up studies (**Civ** and **Cv**).

**Figure 5:**
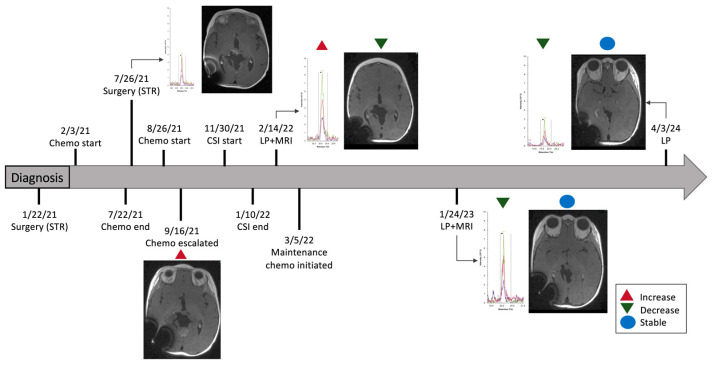
Integrated clinical timeline of treatment, CSF proteomic surveillance, and MRI findings in patient 1715.

In contrast, peptide GTFADVTQVTSLWLAHNEVR in patient 1715, diagnosed with ATRT, demonstrated a dynamic pattern across four CSF samples collected over nearly three years. Diagnosis occurred on DOL 68 following subtotal resection of the tumor. Chemotherapy was administered on DOL 80–249. Repeat surgical resection was performed on DOL 253 with residual tumor identified on postoperative imaging. The first surveillance CSF sample analyzed on MRM was obtained on DOL 253. PAR increased from 0.50 ± 0.09 on DOL 253 to 1.24 ± 0.13 on DOL 456. During this time, the patient received another chemotherapy regimen (DOL 284–305) and CSI (DOL 380–421) due to tumor growth. Imaging initially showed an increase in tumor size on DOL 302, followed by an approximate 80% reduction in tumor volume on MRI performed on DOL 345, and stable tumor size on DOL 353. Subsequent CSF samples after DOL 253 showed a gradual decrease in PAR to 1.13 ± 0.27 on DOL 800 and 0.23 ± 0.18 on DOL 1253 (Figure 4 and Figure 5). This trajectory paralleled a radiographic course showing stable residual enhancement over time while the patient was treated with maintenance chemotherapy. The patient’s most recent imaging on DOL 1507 remained stable, with no new lesions or radiographic evidence of progression.

## 4. Discussion

In this methodological study, we sought to determine whether tumor-specific peptides (TSPs) identified from individual pediatric brain tumors could be detected and longitudinally quantified in the cerebrospinal fluid using a targeted mass spectrometry approach.

Building upon prior proteogenomic discovery methods [40], we applied a streamlined workflow inclusive of tumor-derived peptides and synthetic standard peptide generation with multiple reaction monitoring to evaluate the feasibility of CSF-based disease surveillance. The correlation of endogenous peptide abundance with serial MRI findings in representative cases provides preliminary evidence that this approach may have clinical utility for monitoring treatment response and disease burden.

### 4.1. Overcoming the Limitations of Conventional CSF Protein Biomarkers

Proteomic biomarker discovery in CSF has historically been limited by low-abundance tumor-derived proteins, wide dynamic concentration ranges, and inter-individual variability, all of which complicate reliable detection and quantification. The methods implemented here were specifically designed to address those challenges through increased biological specificity rather than broad discovery alone. By restricting analysis to tumor-derived peptides predicted to be uniquely tumor-specific, we reduced background noise from host proteins and enhanced sensitivity for detecting disease-relevant signals in limited CSF volumes. While other biomarker studies may utilize tissue, serum, or urine samples, CSF was chosen for its quality as a biologically enriched compartment for detecting tumor-derived peptides due to its direct contact with tumor tissue and reduced systemic dilution. As such, CSF is not proposed as universally superior to other biomarkers, but rather as contextually advantageous for longitudinal surveillance in appropriately selected patients.

To detect tumor-specific peptides, we used a two-stage mass spectrometry strategy involving initial LC-MS/MS discovery matched to individualized tumor proteogenomic databases, followed by synthesis of isotopically labeled standards and quantitative MRM in serial CSF samples. This staged approach overcomes the challenges associated with low-abundance biomarkers in CSF and mirrors the success seen in other oncologic studies [42,43].

The advent of mass spectroscopy machines with high mass accuracy in recent years allows for more sensitive peptide detection and relative quantitation. As the methods described within this study also use commercially available reagents and explicitly defined conditions, the protocol may be replicated across centers, addressing a major obstacle to cross-study comparison and large-scale validation in proteomic CSF biomarker research.

### 4.2. Tumor-Specific Peptide Discovery

Using stringent, high-confidence criteria, we identified 33 TSPs across six pediatric brain tumor samples. Notably, some TSPs detected in CSF did not appear in the corresponding tumor samples. This potentially reflects a subset of peptides that are secreted or shed by the tumor into the CSF, highlighting the value of CSF-based biomarker detection given its direct contact with the tumor microenvironment. It is also important to note that, unlike genomic sequencing, where variant absence is typically interpreted as true, missing values in proteomic analysis do not necessarily equate to biological absence and more likely reflect technical under-sampling inherent to data-dependent mass spectrometry.

### 4.3. Temporal Changes in TSP Abundance as Biomarkers of Response

A key contribution of this study is the longitudinal quantification of tumor-specific peptides (TSPs), demonstrating proof-of-concept that dynamic changes in CSF peptide abundance may reflect disease course. In patient 1659, TSP quantity steadily declined in parallel with radiographic improvement and eventual disease resolution, supporting its potential prognostic value. Conversely, in patient 1715, an early rise in TSP abundance mirrored interval tumor progression, followed by a gradual decline that tracked with subsequent treatment response. The delayed normalization of peptide levels compared with rapid radiographic improvement raises two possibilities: (1) tumor cell death may release TSPs in greater quantities that persist for some period of time, or (2) TSPs may capture residual disease that imaging alone does not detect. Blood–brain barrier integrity may also influence the detection of tumor-derived peptides in CSF. Surgical manipulation, radiation therapy, or treatment-related inflammation could alter permeability and thereby affect peptide release independent of tumor burden, a factor that was not systematically evaluated in this study. Disambiguating the potential of TSPs relative to disease course and recurrence prediction will require larger longitudinal cohorts with standardized sampling and outcome correlation. Nevertheless, these cases illustrate the potential for individualized CSF-based peptide monitoring to complement radiographic surveillance. A critical consideration in interpreting these findings is that TSPs identified in this study are primarily patient-specific rather than disease-specific biomarkers. While a small number of peptides were observed in more than one tumor, the study was not designed to define medulloblastoma- or ATRT-specific peptide signatures. Instead, the results support a model in which patients serve as their own biological controls, with longitudinal changes in individualized TSP abundance reflecting disease dynamics.

These findings align with broader trends in oncology, showing the value of proteomic biomarkers for dynamic disease monitoring [44]. The advantage of utilizing tumor-specific peptides has been supported by the TESTBREAST study, in which longitudinal serum proteomics in high-risk women demonstrated that most proteins exhibit remarkable intrapatient stability but large interpatient differences, with strong clustering of serial samples from the same individual over time [45]. This pattern supports the concept that patients can serve as their own controls and that small, individualized deviations in protein abundance may indicate early tumor development or progression. Together, these results suggest that CSF TSP monitoring may complement current radiographic and cytologic surveillance tools by providing individualized, tumor-derived measurements of disease activity. The estimated turnaround time for development of a patient-specific targeted MS assay following tumor resection is approximately 2–3 months, reflecting the time required for proteogenomic analysis and assay optimization. As routine surveillance imaging typically occurs several months after surgery and often later in the treatment course, this approach is most appropriately positioned as a tool for longitudinal disease monitoring rather than immediate post-operative decision-making. Continued longitudinal and multi-omic validation will be essential to establish TSP-based assays as reliable decision-support tools in pediatric neuro-oncology.

### 4.4. Complementarity of TSP Biomarkers with Cellular Immunotherapy

The overwhelming majority of tumor-specific peptides are not derived from functional proteins but rather are sourced from translation of aberrant genomic events resulting from impaired DNA repair and non-canonical pre-mRNA splicing. In this work, we have leveraged these peptides as biomarkers, but it is important to note that they can also represent immunogenic epitopes (neoantigens) that can be targeted by T-cell receptor (TCR)-based cellular immunotherapies [46]. Multiple discovery platforms have been developed, including genomic prediction models, personalized peptide database proteogenomics, immunopeptidomics to identify peptides presented by major histocompatibility complexes (MHC), and surfacesome methods to detect membrane-bound targets. When these neoantigen peptides are also used as biomarkers of disease presence, they can perform double duty in the therapeutic context as both targets of therapy and indicators of that therapy’s efficacy.

### 4.5. Limitations

Several limitations warrant discussion. The small cohort size and limited number of patients with longitudinal CSF sampling preclude formal assessment of sensitivity, specificity, or predictive performance. Mass spectrometry-based detection remains constrained by peptide abundance, digestion efficiency, and sampling depth. Analytical validation of individual peptide assays, including reproducibility, stability, and limits of quantification, will be required prior to clinical translation.

Our use of highly stringent criteria to define “high-confidence” tumor-specific peptides, including the requirement for zero post-translational modifications and zero missed cleavages, may have limited the number of candidate peptides advanced from shotgun discovery to targeted analysis. Though this conservative filtering strategy was intentionally employed to prioritize specificity and reproducibility, relaxation of these criteria may yield a larger pool of tumor-derived peptide candidates detectable in CSF. Specificity in this study was achieved through proteogenomic filtering against normal human protein databases and non-tumor tissue references rather than direct comparison to a healthy CSF control cohort. While this approach reduces the likelihood of identifying physiological background proteins, future studies incorporating healthy CSF controls would further strengthen analytical validation.

Additionally, mass spectrometry is inherently optimized for tryptic peptides within a defined size range (6–30 amino acids), which may limit detection of large TSPs. Accordingly, these findings should be interpreted as proof-of-principle rather than definitive evidence of clinical utility.

Of note, the proteogenomic workflow described here is not intended to replace conventional histologic diagnosis. Identification of tumor-specific peptides requires initial access to tumor tissue in order to construct individualized peptide databases. Accordingly, this approach is best conceptualized as a complementary surveillance tool following tissue-confirmed diagnosis, rather than a standalone diagnostic modality or tool for early risk-stratification. Importantly, the tumor-specific peptides identified in this study are primarily individualized, patient-derived biomarkers rather than disease-level signatures shared across tumor types. While a small subset of peptides was observed across multiple cases, the present study was not designed or powered to establish medulloblastoma- or ATRT-specific peptide markers, nor to evaluate histology-dependent differences in peptide shedding or detectability. These factors may influence the generalizability of peptide-based monitoring across tumor subtypes and remain important areas for future investigation. While future advances in shared disease-level peptide discovery may expand applicability, substantial validation would be required before considering replacement of histopathologic or cytologic evaluation.

### 4.6. Emerging Technologies in Protein Biomarker Discovery

Advances in mass spectrometry platforms and artificial intelligence (AI)-driven analytical tools continue to improve peptide biomarker detection in neuro-oncology for use in diagnosis, prognostication, and longitudinal monitoring. A recent systematic review analyzing 141 studies of CSF proteomics in neuro-oncology highlighted that modern mass spectrometry platforms can reliably profile tumor-associated peptides from ultra-low CSF volumes [47]. Recent work has also demonstrated that intracranial CSF proteomics serves as a dynamic reservoir of biomarkers that can evaluate the effects of resection and other therapies over time, with significant differences identified between lumbar, subarachnoid, and ventricular CSF locations [48]. This finding reinforces the importance of standardized sampling when developing longitudinal biomarkers.

Adjunctive tools to traditional LC-MS/MS, such as ion mobility-enabled platforms and data-independent acquisition (DIA) methods, further enhance the detection of disease-specific peptides by reducing stochastic sampling and missing data across samples, addressing a key limitation of traditional discovery proteomics [49,50,51,52]. Front-end innovations, including ion-filtering approaches such as field asymmetric ion mobility spectrometry (FAIMS), further enhance selectivity and signal-to-noise ratio for low-abundance peptides [53,54]. Complementing discovery workflows, targeted peptide enrichment strategies, such as immunoaffinity-based approaches, offer a potential bridge between broad proteomic discovery and ultrasensitive targeted quantification, particularly for low-abundance biomarkers [55,56].

In parallel, AI and machine-learning tools have become increasingly important in proteomics, offering the ability to extract predictive patterns from high-dimensional peptide abundance matrices and to build biomarker panels that outperform single-biomarker approaches. As highlighted in recent high-throughput proteomics studies, coupling MS-based data with AI facilitates automated feature selection, disease classification, and longitudinal response prediction, further expanding the clinical utility of peptide biomarkers in neuro-oncology [57].

## 5. Conclusions

This study demonstrates the feasibility of identifying and quantifying tumor-specific peptides for longitudinal monitoring of pediatric brain tumors through a combined shotgun discovery and subsequent targeted MRM approach. The observed correlation between CSF peptide abundance and radiographic disease course in the two patients presented supports further investigation of this strategy as an individualized biomarker for disease surveillance and treatment response monitoring. The methodology described here provides a strong foundation for larger prospective studies aimed at validating TSPs as biologically relevant biomarkers in pediatric neuro-oncology.

## Figures and Tables

**Table 1:** Results from shotgun analysis of six pediatric brain tumor tissue samples.

Patient ID (PID)	Diagnosis	Unique Peptides	Screened Peptides	Xcorr *>* 2	High-Confidence *
1770	Medulloblastoma	106,959	45	31	4
1758	Medulloblastoma	140,622	92	50	7
583	Medulloblastoma	106,697	64	37	2
1659	Medulloblastoma	133,376	89	41	14
1697	Medulloblastoma	95,772	3	3	1
1715	ATRT	118,797	18	18	5
Total		702,223	311	180	33

* High-confidence peptides were defined as peptides with zero post-translational modifications and zero missed tryptic cleavages.

**Table 2:** Results from shotgun analysis of six CSF samples.

PID	Unique Peptides	Screened Peptides	Xcorr > 2	High-Confidence *
1770	15,454	11	3	0
1758	7041	10	4	1
583	11,322	12	4	0
1659	11,836	14	3	2
1697	9970	14	4	1
1715	14,168	13	4	2
Total	69,791	74	22	6

* High-confidence peptides were defined as peptides with zero post-translational modifications and zero missed tryptic cleavages.

## Data Availability

The data presented in this study are not publicly available due to ethical and privacy restrictions related to patient confidentiality. De-identified data may be made available from the corresponding author upon reasonable request and with appropriate institutional approvals.

## Supplementary Materials

The following supporting information can be downloaded at: https://www.mdpi.com/article/10.3390/cells15050474/s1, Figure S1: Mass spectra of synthetically-produced peptide GTFADVTQVTSLWLAHNEVR from sample 1715 for multiple reaction monitoring; Figure S2: Mass spectra of synthetically-produced peptide SSNSPHSPIVEEQVPYNK from sample. 1659 for multiple reaction monitoring; Table S1: Tumor Specific Peptides Identified in Tumor Tissue and Corresponding CSF Samples.

## Abbreviations

The following abbreviations are used in this manuscript:

AI	artificial intelligence
ATRT	atypical teratoid/rhabdoid tumor
cfDNA	cell-free DNA
CSI	craniospinal irradiation
CSF	cerebrospinal fluid
DDM	n-dodecyl-*β*-D-maltoside
DIA	data-independent acquisition
DOL	day of life
FAIMS	field asymmetric ion mobility spectrometry
FDR	false discovery rate
GTEx	Genotype-Tissue Expression
HCD	higher-energy collisional dissociation
LC-MS/MS	liquid chromatography–tandem mass spectrometry
LLOD	lower limit of detection
MRI	magnetic resonance imaging
MRD	minimal residual disease
MRM	multiple reaction monitoring
MS	mass spectrometry
m/z	mass-to-charge ratio
NCBI	National Center for Biotechnology Information
PAR	peak area ratio
PET	positron emission tomography
PNET	primitive neuroectodermal tumor
PSM	peptide spectral match
QE buffer	quantitative elution buffer
RNA-seq	RNA sequencing
TCEP	tris(2-carboxyethyl)phosphine
TCR	T-cell receptor
TEAB	triethylammonium bicarbonate
TFA	trifluoroacetic acid
TQMS	triple quadrupole mass spectrometer
TSP	tumor-specific peptide
Xcorr	cross-correlation score
