# Supplementary material for: Longitudinal Detection of Tumor-Specific Peptides in Cerebrospinal Fluid for Pediatric Brain Tumor Surveillance
Source: Cells. 2026 Mar 5;15(5):474. doi: 10.3390/cells15050474 (PMC12984984; doi:10.3390/cells15050474)
Supplement: Supplementary file 1 [file cells-15-00474-s001.zip › cells-4124000-supplementary.pdf]

Table S1.cific Peptides Identified in Tumor Tissue and Corresponding CSF Samples

Patient ID (PID)	Peptides Identified	Event Type	Gene/Transcript Label	Coordinates	Variant
1770			Tumor Tissue		
	NVGTSQELLEYETTV	SNV	XKR7	-	S565N
	QLDNILAAVHDVLNESK	JUNC	JUNC00114716	chr16:30298587–30299869	-
	LHELSQFK	FUS	RPS3A_RPL10	-	-
	ISAVSEIQTLGR	JUNC	JUNC00240039	chr4:189726594–189728469	-
			CSF (None)		
1758			Tumor Tissue		
	VHSPAVPIDHSDLVADLLK	JUNC	JUNC00196138	chr2:121365284–121365889	-
	ATSQGGDLMSDLFNK	SNV	ENSP00000485442	-	L19F, Q54R, P320S, M382V, H406D
	GSNPHMQTFTFTR	SNV	ESYT1	-	L176M
	VLSTSTDLEAAVADALKPVR	JUNC	JUNC00196175	chr2:121407712–121410866	-
	TTAEYQLLVEGVPSPR	SNV	PECAM1	-	V125L
	LHELSQFK	FUS	TSN_RPS3A	-	-
	GVVSVEVER	SNV	CEBPZ	-	G422V
			CSF (None)		
583			Tumor Tissue		
	GGPSDHEATAWILGLR	JUNC	JUNC00239996	chr9:38409217–38413237	-
	EAATAADTK	FUS	LGALS1_RPS9	-	-
			CSF		
	TPAPPPTPR	JUNC	JUNC00087266	chr16:4466402-4474093	-
1659			Tumor Tissue		
	SSNSPHSPIVEEFQVPYN	SNV	C1S	-	N90S
	ATSQGGDLMSDLFNK	SNV	ENSP00000485442	-	Q54R, G98E, P119S, P320S, S339G, M382V, H406D
	TVEFQVEELPEVK	JUNC	JUNC00282483	chr7:82062390–82064304	-
	VSAPDPPPPHPAR	FUS	PTMS_ACTG1	-	-
	DLSAMEPNDSTSTAVEEPDSLEVLVK	FUS	BAG6_C6orf47	-	-
	QEKPAPPKPEPKPR	JUNC	JUNC00266962	chr6:79203630–79214972	-
	TGLELLR	JUNC	JUNC00114454	chr16:22292324–22296230	-
	SMYEEINETR	JUNC	JUNC00250656	chr5:126805696–126810183	-
	ESLSDLDTR	FUS	EEF1A1_RPS20	-	-
	PAESQPGGYPAAGHPGR	JUNC	JUNC00141034	chr17:75892539–75892755	-

	LHELSQFK	FUS	RPS3A_ACTG1	-	-
	LEQLGPQAQTTK	FUS	TUBA1B_RPS4X	-	-
	ESLLLGAK	FUS	FN1_HMGN2	-	-
	NVGFLNR	FUS	HNRNPK_RPL13A	-	-
			CSF		
	SSNSPHSPIVEEFQVPYN	SNV	C1S	-	N90S
	GALALLLFAAGGHR	JUNC	JUNC00083263	chr13:49567320-49585444	-
1697			Tumor Tissue		
	LHELSQFK	FUS	SHMT2_EEF1A1	-	-
			CSF		
	LLDYHLNQPFIFVLR	SNV	SERPINF1	-	P385L
1715			Tumor Tissue		
	GGAAPAGGGAEAGPGGGPGGAGGAAAK	FUS	ATP9A_EEF1A1	-	-
	TGLDSPTGIDFSDITANYFTVHWIAPR	SNV	FN1	-	S1464Y
	ATSQGGDLMSDLFNK	SNV	WASH1	-	F19L, D141Y, Q392K, H407D
	FLAPFTAHPLDGGR	SNV	TMEM132A	-	A556T
	IGANTSTQTMGPRPAAAAAAATPAVR	FUS	EEF1A1_PABPC1	-	-
			CSF		
	GTFADVTQVTSLWLAHNEVR	SNV	ISLR2	-	A70T
	TGLDSPTGIDFSDITANYFTVHWIAPR	SNV	FN1	-	S1464Y

Origin abbreviations: SNV, single nucleotide variant; JUNC, tumor-specific novel splice junction; FUS, gene-fusion derived. Variant column reflects amino acid substitutions when present in proteogenomic mapping. Full accession identifiers are available upon request.

Patient ID (PID)	Peptides Identified	Event Type	Gene/Transcript Label	Coordinates	Variant
1770			Tumor Tissue		
	NVGTSQELLEYETTV	SNV	XKR7	-	S565N
	QLDNILAAVHDVLNESK	JUNC	JUNC00114716	chr16:30298587–30299869	-
	LHELSQFK	FUS	RPS3A_RPL10	-	-
	ISAVSEIQTLGR	JUNC	JUNC00240039	chr4:189726594–189728469	-
			CSF (None)		
1758			Tumor Tissue		
	VHSPAVPIDHSDLVADLLK	JUNC	JUNC00196138	chr2:121365284–121365889	-
	ATSQGGDLMSDLFNK	SNV	ENSP00000485442		L19F, Q54R, P320S, M382V, H406D
	GSNPHMQTFTFTR	SNV	ESYT1	-	L176M
	VLSTSTDLEAAVADALKPVR	JUNC	JUNC00196175	chr2:121407712–121410866	-

	TTAEYQLLVEGVPSPR	SNV	PECAM1	-	V125L
	LHELSQFK	FUS	TSN_RPS3A	-	-
	GVVSVEVER	SNV	CEBPZ	-	G422V
	CSF (None)				
583	Tumor Tissue				
	GGPSDHEATAWILGLR	JUNC	JUNC00239996	chr9:38409217–38413237	-
	EAATAADTK	FUS	LGALS1_RPS9	-	-
	CSF				
	TPAPPPTPR	JUNC	JUNC00087266	chr16:4466402-4474093	-
1659	Tumor Tissue				
	SSNSPHSPIVEEFQVPYN	SNV	C1S	-	N90S
	ATSQGGDLMSDLFNK	SNV	ENSP00000485442	-	Q54R, G98E, P119S, P320S, S339G, M382V, H406D
	TVEFQVEELPEVK	JUNC	JUNC00282483	chr7:82062390–82064304	-
	VSAPDPPPPHPAR	FUS	PTMS_ACTG1	-	-
	DLSAMEPNDSTSTAVEEPDSLEVLVK	FUS	BAG6_C6orf47	-	-
	QEKPAPPKPEPKPR	JUNC	JUNC00266962	chr6:79203630–79214972	-
	TGLELLR	JUNC	JUNC00114454	chr16:22292324–22296230	-
	SMYEEINETR	JUNC	JUNC00250656	chr5:126805696–126810183	-
	ESLSDLDTR	FUS	EEF1A1_RPS20	-	-
	PAESQPGGYPAAGHPGR	JUNC	JUNC00141034	chr17:75892539–75892755	-
	LHELSQFK	FUS	RPS3A_ACTG1	-	-
	LEQLGPQAQTTK	FUS	TUBA1B_RPS4X	-	-
	ESLLLGAK	FUS	FN1_HMGN2	-	-
	NVGFLNR	FUS	HNRNPK_RPL13A	-	-
	CSF				
	SSNSPHSPIVEEFQVPYN	SNV	C1S	-	N90S
	GALALLLFAAGGHR	JUNC	JUNC00083263	chr13:49567320-49585444	-
1697	Tumor Tissue				
	LHELSQFK	FUS	SHMT2_EEF1A1	-	-
	CSF				
	LLDYHLNQPFIFVLR	SNV	SERPINF1	-	P385L
1715	Tumor Tissue				
	GGAAPAGGGAEAGPGGGPGGAGGAAAK	FUS	ATP9A_EEF1A1	-	-
	TGLDSPTGIDFSDITANYFTVHWIAPR	SNV	FN1	-	S1464Y
	ATSQGGDLMSDLFNK	SNV	WASH1	-	F19L, D141Y, Q392K, H407D

	FLAPFTAHPLDGGR	SNV	TMEM132A	-	A556T
	IGANTSTQTMGPRP AAAAAAATPAVR	FUS	EEF1A1_PABPC1	-	-
			CSF		
	GTFADVTQVTSLW LAHNEVR	SNV	ISLR2	-	A70T
	TGLDSPTGIDFSDIT ANYFTVHWIAPR	SNV	FN1	-	S1464Y

Origin abbreviations: SNV, single nucleotide variant; JUNC, tumor-specific novel splice junction; FUS, gene-fusion derived. Variant column reflects amino acid substitutions when present in proteogenomic mapping. Full accession identifiers are available upon request.

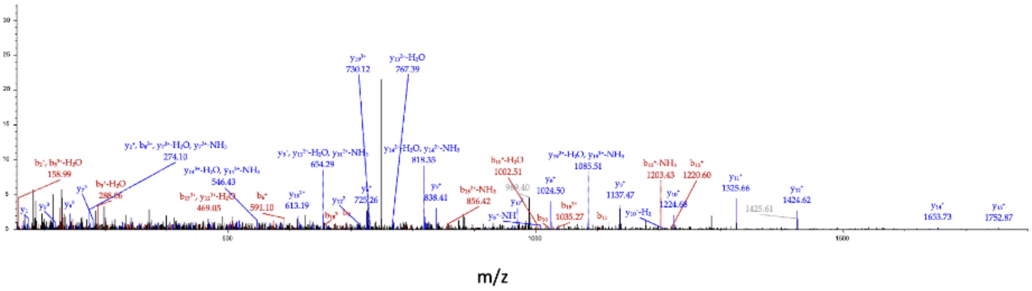

Figure S1. Mass spectra of synthetically-produced peptide GTFADVTQVTSLWLAHNEVR from sample 1715 for multiple reaction monitoring

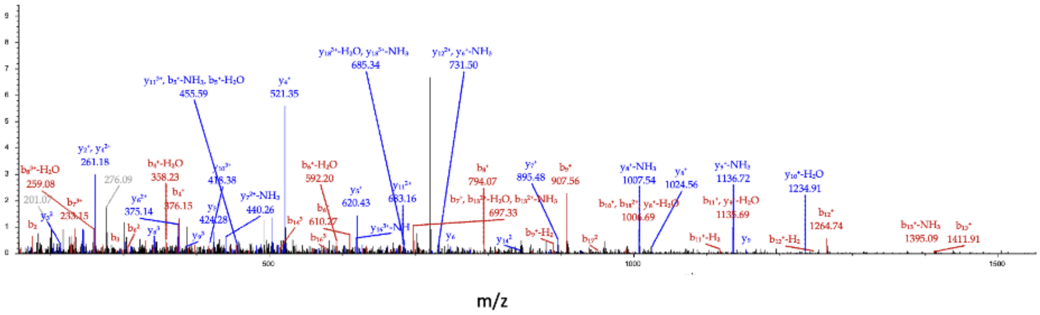

Figure S2. Mass spectra of synthetically-produced peptide SSNSPHSPIVEEQVPYNK from sample 1659 for multiple reaction monitoring
